# Construction and experimental validation of a signature for predicting prognosis and immune infiltration analysis of glioma based on disulfidptosis-related lncRNAs

**DOI:** 10.3389/fimmu.2023.1291385

**Published:** 2023-11-03

**Authors:** Youwei Guo, Zhipeng Jiang, Quan Chen, Dongcheng Xie, Yi Zhou, Wen Yin, Zihan Wang, Binbin Wang, Caiping Ren, Xingjun Jiang

**Affiliations:** ^1^ Department of Neurosurgery, Xiangya Hospital, Central South University, Changsha, Hunan, China; ^2^ National Clinical Research Center for Geriatric Disorders, Xiangya Hospital, Central South University, Changsha, Hunan, China; ^3^ Department of Neurosurgery, the First Affiliated Hospital with Nanjing Medical University, Nanjing, China; ^4^ Cancer Research Institute, Department of Neurosurgery, National Clinical Research Center for Geriatric Disorders, Xiangya Hospital, Central South University, Changsha, Hunan, China; ^5^ National Health Commission (NHC) Key Laboratory of Carcinogenesis and the Key Laboratory of Carcinogenesis and Cancer Invasion of the Chinese Ministry of Education, School of Basic Medical Science, Central South University, Changsha, Hunan, China

**Keywords:** glioma, lncRNA, disulfidptosis, immune therapy, bioinformatics

## Abstract

**Backgrounds:**

Disulfidptosis, a newly discovered mechanism of programmed cell death, is believed to have a unique role in elucidating cancer progression and guiding cancer therapy strategies. However, no studies have yet explored this mechanism in glioma.

**Methods:**

We downloaded data on glioma patients from online databases to address this gap. Subsequently, we identified disulfidptosis-related genes from published literature and verified the associated lncRNAs.

**Results:**

Through univariate, multivariate, and least absolute shrinkage and selection operator (LASSO) regression algorithms analyses, we identified 10 lncRNAs. These were then utilized to construct prognostic prediction models, culminating in a risk-scoring signature. Reliability and validity tests demonstrated that the model effectively discerns glioma patients’ prognosis outcomes. We also analyzed the relationship between the risk score and immune characteristics, and identified several drugs that may be effective for high-risk patients. In vitro experiments revealed that LINC02525 could enhances glioma cells’ migration and invasion capacities. Additionally, knocking down LINC02525 was observed to promote glioma cell disulfidptosis.

**Conclusion:**

This study delves into disulfidptosis-related lncRNAs in glioma, offering novel insights into glioma therapeutic strategies.

## Introduction

Glioma, originating from neuroepithelial cells, represents the most prevalent malignant tumor in the central nervous system (CNS) (1), accounting for approximately 70% of intracranial malignant tumors (2). Glioma has a global annual incidence of 4-6 per 100,000 (3) individuals and is responsible for up to 30,000 deaths annually. However, patients diagnosed with glioblastoma multiforme (GBM) face a bleak prognosis, characterized by a median survival time of just 12-15 months and a 5-year survival rate of<5% (4). While the combination of surgery, radiotherapy, and chemoradiotherapy remains the primary treatment for glioma (5), it is imperative to delve deeper into glioma pathogenesis and identify potent therapeutic targets.

Since 2007, the classification of CNS tumors has incorporated molecular diagnosis into its criteria (6). The most recent classification integrates factors such as IDH mutation, 1p/19q codeletion, ATRX and/or TP53 mutations, and MGMT promoter methylation, thus forming a new tissue-molecular classification (7). IDH mutation status is a decisive marker for the classification and prognosis assessment of gliomas, with all wild-type IDH gliomas classified as WHO grade 4, emphasizing its importance for glioma diagnosis and prognosis. Although these classifications, combined with other therapies (8, 9), guide clinicians in diagnosing and treating gliomas, effective therapeutic targets are still lacking. It’s pivotal to further investigate the molecular changes involved in their development to improve the prognosis of gliomas.

As bioinformatics and functional genomics have advanced, researchers have established several pivotal cancer research databases, including The Cancer Genome Atlas (TCGA), Gene Expression Omnibus (GEO), and Chinese Glioma Genome Atlas (CGGA) (10–12). These databases have allowed researchers to identify potential tumorigenic genes and facilitate the search for glioma-related molecular targets.

Apoptosis, an essential cell death mechanism, can be initiated both internally and externally. This process activates caspases, leading to the cleavage of vital proteins, resulting in cell death (13). Recent findings suggest multiple cell death mechanisms, including ferroptosis, pyroptosis, anoikis, and cuproptosis, play roles in tumor progression (14–17). Among these, “disulfidptosis” stands out as it is linked to the actin cytoskeleton, an essential cellular framework ensuring cell shape and survival. This cytoskeleton is built from actin filaments, fundamental for cellular shape and structure. Disulfide stress caused by increased cystine intake and a lack of NADPH, can destabilize the actin structure, potentially causing cell death if left untreated (18). These observations underscore the importance of irregular disulfide bonds in cancer evolution and offer valuable perspectives for treating aggressive cancers, notably gliomas.

Long non-coding RNAs (lncRNAs) are gene expression regulators that comprise more than 200 nucleotides and lack protein-coding potential, are involved in the cell cycle, cellular invasion, and immune response during pathophysiological processes (19, 20). There has been extensive evidence that lncRNAs play a role in the progression of many malignant tumors, including gliomas (21), which makes them promising as novel biomarkers and therapeutic targets for cancer (22–24). Signatures based on lncRNA have been proven effective in accurately evaluating the survival status of patients with most tumors (25, 26). Therefore, investigating the role of disulfidptosis-related lncRNAs (DRlncRNAs) in glioma is crucial.

Our study analyzed the 10 known genes associated with disulfidptosis on a pan-cancer basis to explore their common features and specificities across different cancers. We then verified the expression of these 10 genes in glioma through immunohistochemistry. Finally, we developed a prognostic signature based on DRlncRNAs and evaluated the gene mutation and tumor immunity in glioma. According to our findings, disulfidptosis may play an important role in the development of gliomas, opening up new therapeutic possibilities.

## Materials and methods

### Dataset and source

**Figure 1:**
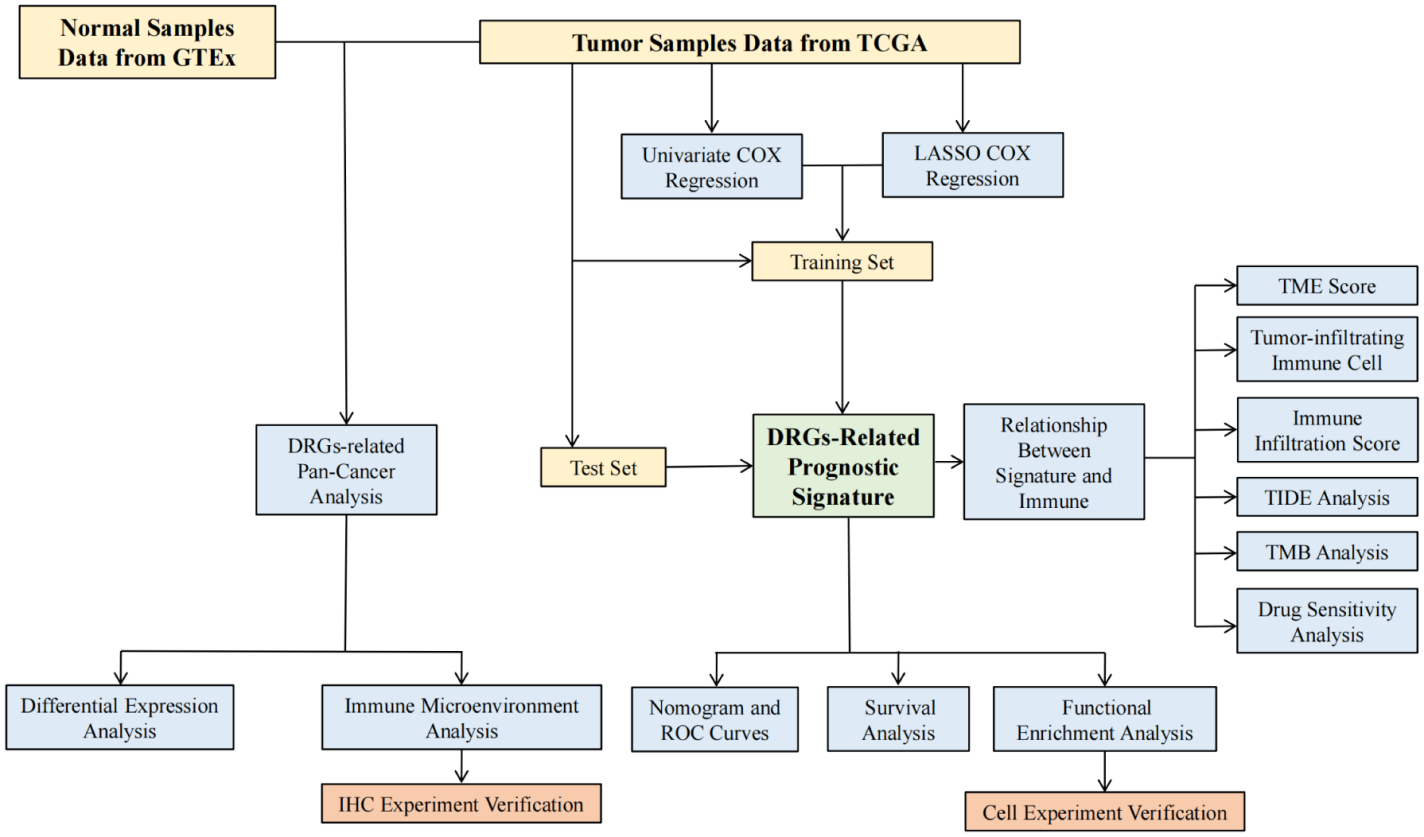
The flow chart of research design.

Ten disulfidptosis-related genes (*NCKAP1*, *SLC7A11*, *NUBPL*, *SLC3A2*, *RPN1*, *NDUFA11*, *GYS1*, *NDUFS1*, *OXSM*, *LRPPRC*) were selected based on previous studies (18, 27, 28). The mRNA and lncRNA expression data and corresponding clinical data of 707 glioma patients were retrieved from the TCGA data portal (https://portal.gdc.cancer.gov/). Part of the molecular subtype information was collected in UCSC Xena (http://xena.ucsc.edu/). The downloaded data were consolidated with the Strawberry Perl program. All data were collated and merged through R 4.2.2. The flowchart of the article design was shown in **Figure 1**.

### Pan-cancer analysis

Pan-cancer analysis of 10 genes was performed using the online tool SANGERBOX 3.0 (http://sangerbox.com/home.html). The differential expression information of genes across 34 tumor types was integrated with TCGA and GTEx databases. These tumor types included GBM, GBMLGG, LGG, UCEC, BRCA, CESC, LUAD, ESCA, STES, KIRP, KIPAN, COAD, COADREAD, PRAD, STAD, HNSC, KIRC, LUSC, LIHC, WT, SKCM, BLCA, THCA, READ, OV, PAAD, TGCT, UCS, ALL, LAML, PCPG, ACC, KICK, and CHOL (**Table S1**). StromalScore, ImmuneScore, and ESTIMATEScore were calculated using the Pearson coefficient. All samples were sourced from the TCGA database; samples with expression values recorded as “0” were excluded.

### Acquisition of immunohistochemical staining information

The data on protein expression for the 10 specified genes in gliomas was retrieved from the Human Protein Atlas website (http://www.proteinatlas.org/). We searched for the 10 genes related to disulfidptosis. However, information was only available for eight genes, including *GYS1*, *SLC3A2*, and *NUBPL*. We then compared samples from healthy tissue, low-grade glioma, and high-grade glioma. The same genes were stained with the same antibody.

### Glioma and disulfidptosis-related genes

The presence of these 10 DRGs in glioma with various immune cells was calculated with the Cibersort algorithm. We used R to achieve a more refined comparison of expression between normal and tumor samples, integrating data from both the GTEx and TCGA databases. The hazard ratio of overall survival for the 10 DRGs in glioma was visualized using the forest plot.

### Development and evaluation of the DRlncRNAs-related prognostic signature

The criteria for selecting DRlncRNAs were established with CorFilter= set at 0.3 and pvalueFilter= at 0.001. We visualized the correlation between 10 DRGs and the selected DRlncRNAs using a Sankey diagram.

In R, the “Survival”, “caret”, “glmnet”, “survminer” and “timeROC” packages were installed and utilized for analyses. We allocated 50% of the sorted cohort data to the training set and the remaining 50% to the test set. Given the sizable gene count, the univariate Cox analysis had a filtering condition set to “coxPfilter = 0.001”. The DRlncRNAs data were analyzed by Lasso regression and cross-validation to select the most appropriate DRlncRNAs combination with the smallest error. Finally, the risk scores of the training set and test set were calculated through the risk score calculation formula:


$$\text{Risk score}=\sum_{i=1}^{n}\left(\beta_{i}\times x_{i}\right)$$

The difference in *P*-value was obtained by comparing the survival differences of two different risk groups. Then, we calculated the area under the ROC curve by evaluating the accuracy of the modified model. The established risk signature was saved if the following conditions were met, given a prediction time of 1 year: *P*-value-train< 0.01, ROC-AUC-train > 0.68, *P*-value-test< 0.05, and ROC-AUC-test > 0.65.

### Prognostic value of the DRlncRNAs-related prognostic signature

Patient risk scores were calculated and reordered using the risk score formula, and the risk heat map was generated using the “Pheatmap” package. Overall survival (OS) and progression-free survival (PFS) analyses were performed for each group.

Clinicopathological characteristics, such as age, grade, and 1p/19q, were used to divide patients into different subgroups. Box plots were drawn by the “ggpubr” and “limma” packages to analyze whether clinical characteristics were associated with risk scores. Subsequently, univariate and multivariate Cox regression analyses were performed using the “survival” package for risk scores and corresponding clinical variables, including age, gender, and grade.

A nomogram was constructed using all independent prognostic factors, including risk score, to predict the probability of OS at 1, 3, and 5 years. The “RMS” package was used to calculate the nomogram’s concordance index (C-index) to evaluate its discriminant ability. Then, ROC curves and decision curves were drawn to evaluate the prognostic prediction accuracy of the risk score and nomogram. Finally, to plot the concordance index, we utilized packages including “dplyr”, “survival”, “rms”, and “pec” in R.

### Principal component analysis

Principal component analysis (PCA) of disulfidptosis was performed using the “Scatterplot3d” package, with results visualized in three dimensions.

### Functional analysis of significant risk differentially expressed genes

The “ClusterProfile” package in R software was used to conduct Gene Ontology (GO) enrichment analysis and Kyoto Encyclopedia of Genes and Genomes (KEGG) pathway analysis on the differentially expressed genes. Statistical significance was set at *P*< 0.05. Gene Set Enrichment Analysis (GSEA) was performed to determine the enrichment of functions or pathways between two groups and the five most significant pathways were displayed.

### Correlation analysis between DRlncRNAs-related prognostic signature and immunity

StromalScore, ImmuneScore, and ESTIMATEScore were calculated using the “estimate” packages, and the scores of the tumor microenvironment were then compared between the two groups. The relative percentage of each type of immune cell and the immune function score were calculated using the “limma”, “reshape2”, “ggpubr”, “GSVA”, and “GSEABase” packages. Patient response was also predicted using the Tumor Immune Dysfunction and Exclusion (TIDE) analysis. The TIDE score was calculated using the online tool available at http://tide.dfci.harvard.edu/, and results were visualized using a violin plot.

### Tumor mutation burden and drug sensitivity analysis

Once downloaded from TCGA, the tumor mutation data were processed and analyzed using the “Perl” programming language. Kaplan-Meier survival curves were plotted to analyze the combined effects of high versus low mutation burden and high- versus low- risk groups. The sensitivity of various drugs’ response to glioma was predicted with “oncoPredict” and “parallel” packages.

### Cell culture

The Key Laboratory of Carcinogenesis and Cancer Invasion of the Chinese Ministry of Education of Central South University, Changsha, China, provided glioma cell lines (T98G and U251). T98G and U251 cells were cultured in high-glucose DMEM (Gibco) containing 10% fetal bovine serum. The siRNAs against the *LINC02525* gene were purchased from RiboBio Corporation (Guangzhou, China).

### Wound-healing assay

Glioma cells from various culture models were seeded onto 6-well plates. Once they achieved 95% confluence, a P-200 pipette tip was used to inflict wounds on the cell monolayers. Subsequently, these wounded layers were gently rinsed thrice with PBS; A medium enriched with 2% FBS was then introduced, followed by plate incubation. Imaging was conducted immediately (0 h) and after 48 hours. During analysis, distances between wound edges were measured at three distinct positions over the specified time intervals.

### Cell migration and invasion assay

Transwell assays were used to detect cell migration and invasion abilities. Transfected U251 or T98G cells (1 × 10^4^ cells) were harvested after 24-48 h transfection and resuspended in 100 μL serum-free medium for cell migration detection. The cells were then seeded into the upper chamber of a Transwell assay insert (Millipore), and 700 μL 10% FBS medium was added to the lower chamber. After incubation at 37 °C for 48 h, the cells on the lower side were washed three times with PBS, fixed in 4% paraformaldehyde for 20 min, and stained with crystal violet solution for 15 min. Cells from five random fields were counted under an inverted microscope (Olympus) for statistical analysis, and photographs were captured. Transwell chambers were coated with Matrigel for 1 h at 37 °C for the invasion assay. The transfected cells (1 × 10^4^ cells) were resuspended in 100 μL serum-free medium and seeded into the upper chamber. Then 700 μL 10% FBS medium was added to the lower chamber. After a 48 hours incubation period, the invasive ability was evaluated, as mentioned previously, for the cell migration assay.

### Immunohistochemistry

The paraffin-embedded tissue microarray of GBM was obtained from the Neurosurgery department of Xiangya Hospital. The GBM tumor tissue (n = 83) and para-tumor tissue (n = 12) were collected from patients who received primary and curative resection without systematic anticancer treatment in our hospital. The consent procedures for human tissues and the protocols were approved by the Ethics Committee of Xiangya Hospital (NO.202307161). IHC assays were performed with the primary antibody against *CD163* (dilution at 1:1000, 16646-1-AP, Proteintech, China), *RPN1* (dilution at 1:200, 12894-1-AP, Proteintech, China), and *GYS1* (dilution at 1:200, 10566-1-AP, Proteintech, China). Microscopic images were captured for calculating IHC scores. The IHC scores were obtained according to the following formula: overall score = percentage score (≤25%, 1; 26–50%, 2; 51–75%, 3; and >75%, 4) × intensity score (no staining, 0; light brown, 1; moderate brown, 2; and deep brown, 3). The scoring was performed in a double-blinded manner by two pathologists.

### Fluorescent staining of actin filaments

The treated U251 cells were cultured in a complete DMEM medium overnight and then treated with low-sugar RPMI-1640 medium for 6 hours. Following this, the cells in the six-well plates were washed three times with PBS and then fixed with a 3.7% formaldehyde solution (prepared in PBS) at room temperature for approximately 10 minutes. After the staining incubation, the cells were washed 2 to 4 times with 0.1% Triton X-100, with each wash lasting 5 minutes. Actin-Tracker Red was used for staining and diluted in a 1:100 ratio with 1% BSA and 0.1% Triton X-100. To each well, 1 mL of the dye solution was added, followed by incubation at room temperature away from light for 30-60 minutes. Next, the cells were washed twice, the nuclei were stained with DAPI, and images of the cells were captured using a microscope.

### Statistical analysis

All statistical data were analyzed using the R statistical language (version 4.2.2). Survival curves were analyzed using the Kaplan-Meier method, and log-rank tests compared the survival between subgroups. For comparisons between two groups based on clinicopathologic features such as age (≤ 65 *vs*. > 65 years old), WHO grade (2 *vs*. 3 *vs*. 4), gender (male *vs*. female), 1p/19q status (codeletion *vs*. non-codeletion), IDH status (wild *vs*. mutation), and ATRX status (wild *vs*. mutation). Student’s t-tests were used. Student’s t-tests were also used to compare experimental groups with the control group in transwell and wound healing assays. *P*-value of< 0.05 was set as the threshold for statistical significance for all analyses. ^ns^ *P* ≥ 0.05, * *P*< 0.05, ** *P*< 0.01, *** *P*< 0.001, ****P< 0.0001.

## Results

### Pan-cancer analysis of ten disulfidptosis-related genes

**Figure 2:**
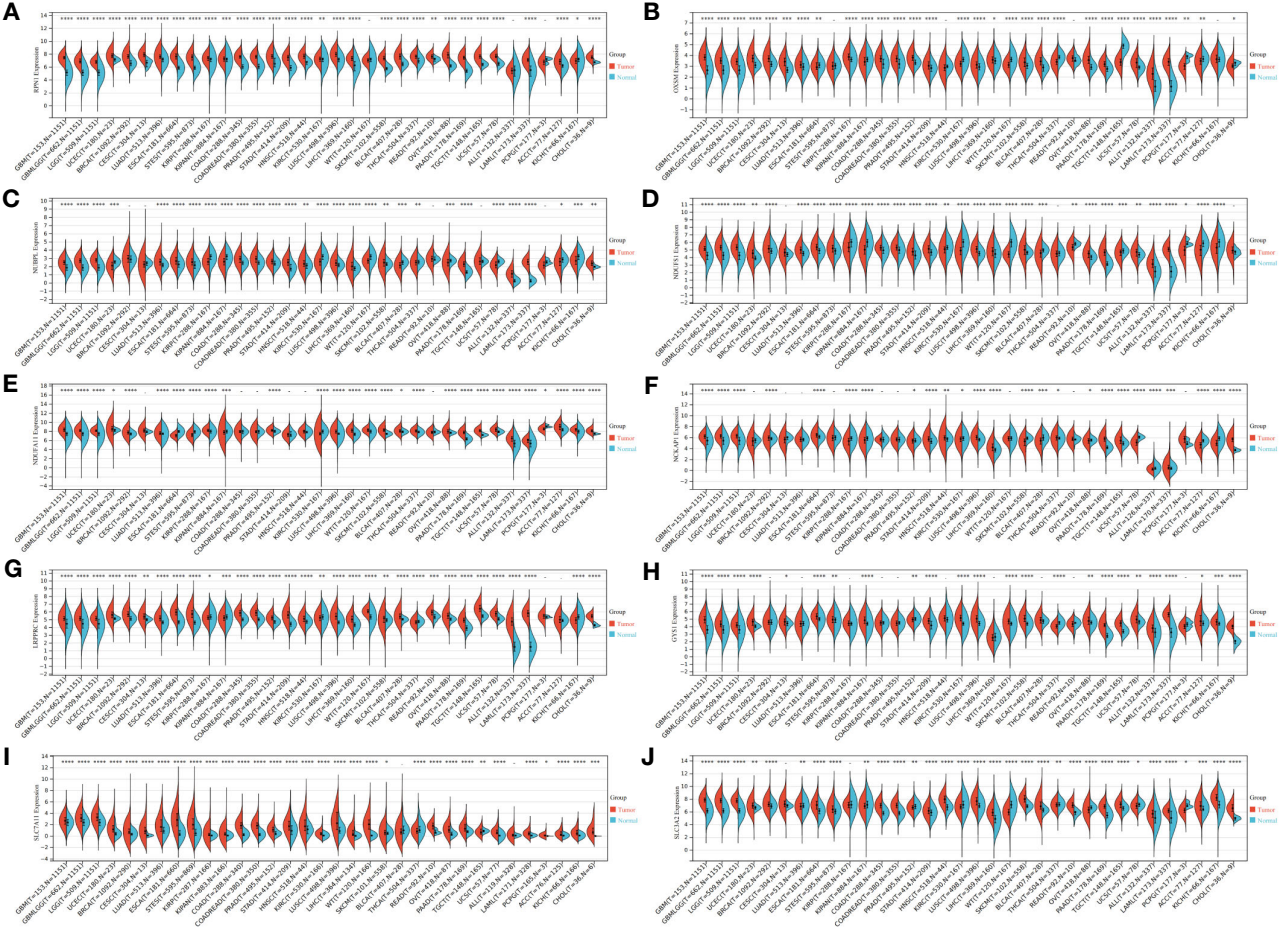
Expression levels of disulfidptosis-related genes in different tumors and normal tissues. **(A–J)** Expression levels of *RPN1*, *OXSM*, *NUBPL*, *NDUFS1*, *NDUFA11*, *NCKAP1*, *LRPPRC*, *GYS1*, *SLC7A11*, and *SLC3A2* in 34 kinds of tumors and corresponding normal tissues in TCGA and GTEx databases. *P < 0.05, **P < 0.01, ***P < 0.001 and ****P < 0.0001.

**Figure 3:**
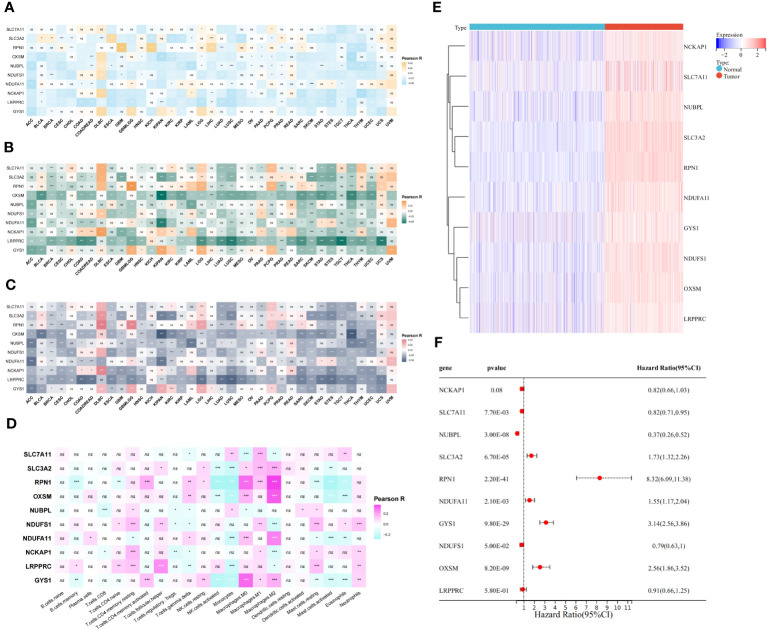
Relationship between disulfidptosis-related genes and glioma immunity and prognosis. **(A–C)** The assessment of TME-related scores between high- and low-risk groups. The relationship between disulfidptosis-related genes and ImmuneScore **(A)**, StromalScore **(B)**, and ESTIMATEScore **(C)**. **(D)** CIBERSORT algorithm showed the correlation analysis between disulfidptosis-related genes and 25 kinds of immune cells in glioma. **(E)** The heat map showed that disulfidptosis genes were differentially expressed in glioma and normal brain tissue and were highly expressed in tumors. **(F)** The forest map showed the results of univariate COX regression analysis between disulfidptosis-related genes and the prognosis of glioma patients. ns(no significance) *P* ≥ 0.05, *P < 0.05, **P < 0.01 and ***P < 0.001.

**Figure 4:**
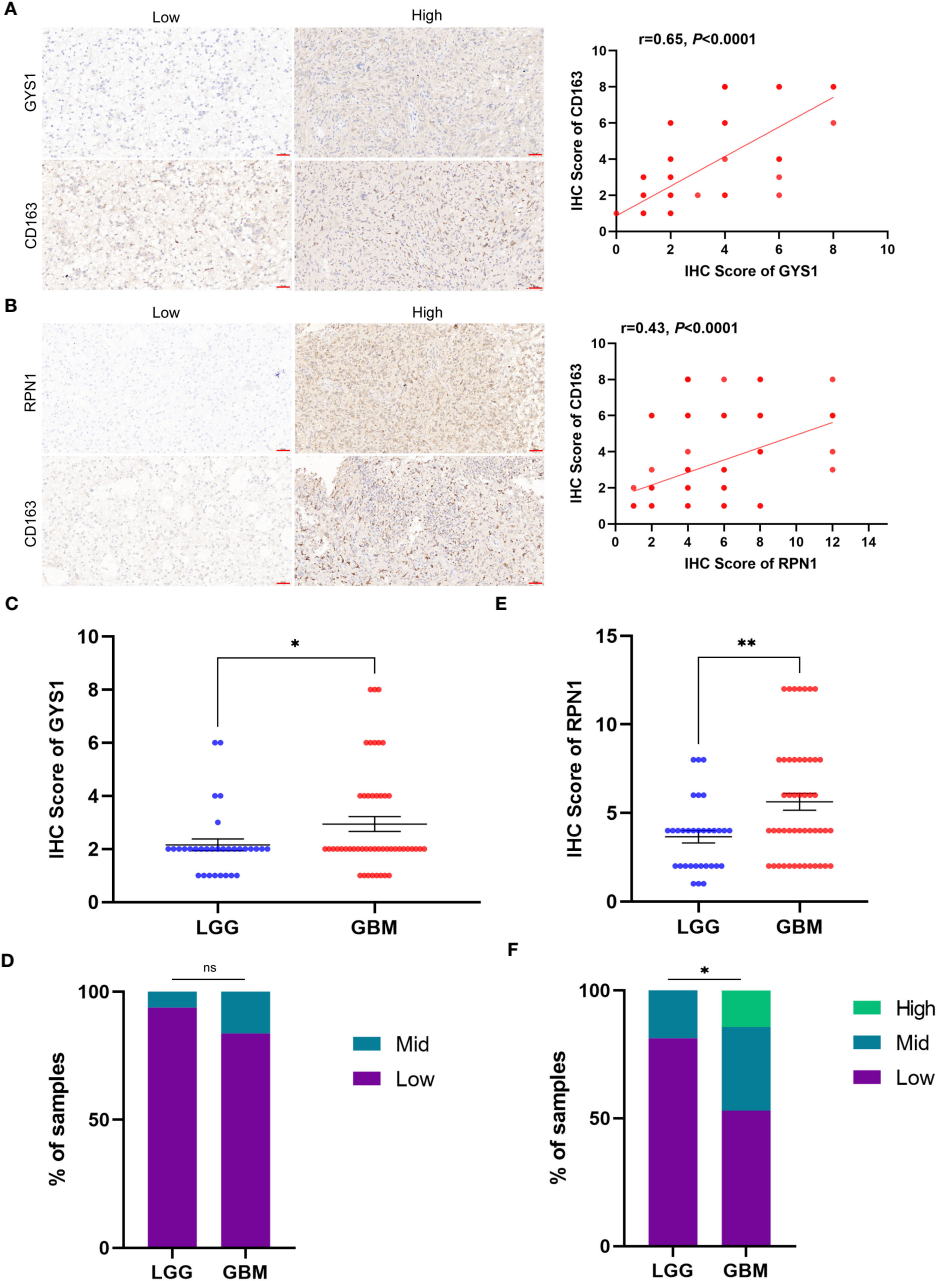
The relationship of protein expression levels between disulfidptosis-related genes and M2 macrophage marker CD163 in glioma tissue samples. **(A)** IHC showed that glioma samples with high *GYS1* expression had a higher expression level of CD163 (M2 macrophage marker). The figure on the right shows the *Pearson correlation analysis* results of IHC scores of *GYS1* and CD163 in tissue samples (r=0.65, *P*<0.0001). **(B)** IHC showed that glioma samples with high *RPN1* expression had a higher expression level of CD163 (M2 macrophage marker). The figure on the right shows the *Pearson correlation analysis* results of IHC scores of *RPN1* and CD163 in tissue samples (r=0.65, *P*<0.0001). **(C, D)** The expression levels of *GYS1* among LGG and GBM. **(E, F)** The expression levels of *RPN1* among LGG and GBM. ns (no significance) *P* ≥ 0.05, *P < 0.05, and **P < 0.01.

We initiated our investigation by analyzing the differential expression of the 10 disulfidptosis-related genes in 34 tumor types. In most tumors, including LGG, GBM and LGGGBM, these DRGs exhibited abnormally high expression compared to normal samples (**Figure 2**). Furthermore, we analyzed the correlation between the expression of 10 DRGs and StromalScore, ImmuneScore, and ESTIMATEScore in 32 tumors. We found a significant positive correlation between the expression of several DRGs (*RPN1*, *GYS1*) and StromalScore (**Figure 3A**), ImmuneScore (**Figure 3B**), and ESTIMATEScore (**Figure 3C**) in glioma. To further understand disulfidptosis in gliomas, we analyzed correlations with various immune cells using the TCGA database. *SLC3A2*, *RPN1*, *OXSM*, *NDUFA11*, and *GYS1* showed a relatively strong positive correlation with macrophages M2 (**Figure 3D**). By integrating the TCGA and GTEx databases, we found that these 10 genes were significantly highly expressed in glioma (**Figure 3E**). The forest map shows that these five genes *(SLC3A2*, *RPN1*, *NDUFA11*, *GYS1*, *OXSM*) might be high-risk factors for glioma prognosis (**Figure 3F**). We also examined *RPN1*, *GYS1*, and *CD163* (M2 macrophage marker) expression levels in 83 glioma tissues. As depicted in **Figures 4A**, **4B**, glioma tissues with increased *GYS1* or *RPN1* expression also exhibited elevated *CD163* expression, aligning with our pan-cancer analysis findings (**Figure 3D**). Subsequent assessment of *GYS1* and *RPN1* expressions within LGG and GBM showed that *GYS1* (**Figures 4C, D**) and *RPN1* (**Figures 4E, F**) displayed higher IHC scores in GBM. Furthermore, based on IHC scores, we classified the samples into three groups: Low (0-4 score), Mid (5-8 score), and High (9-12 score). The results showed that the proportion of Mid and High IHC scores of *RPN1* (**Figure 4F**, *P<* 0.05) in GMB was higher.

Moreover, we also examined the expression profiles of these ten DRGs using Protein Atlas data. The data underscored that *GYS1*, *SLC3A2*, *NUBPL*, and *PRN1* were predominantly expressed in tumors, and notably, tissues with a higher grade displayed significantly increased these protein expressions of these genes (**Figure S1**).

### Construction and assessment of the risk score signature

**Figure 5:**
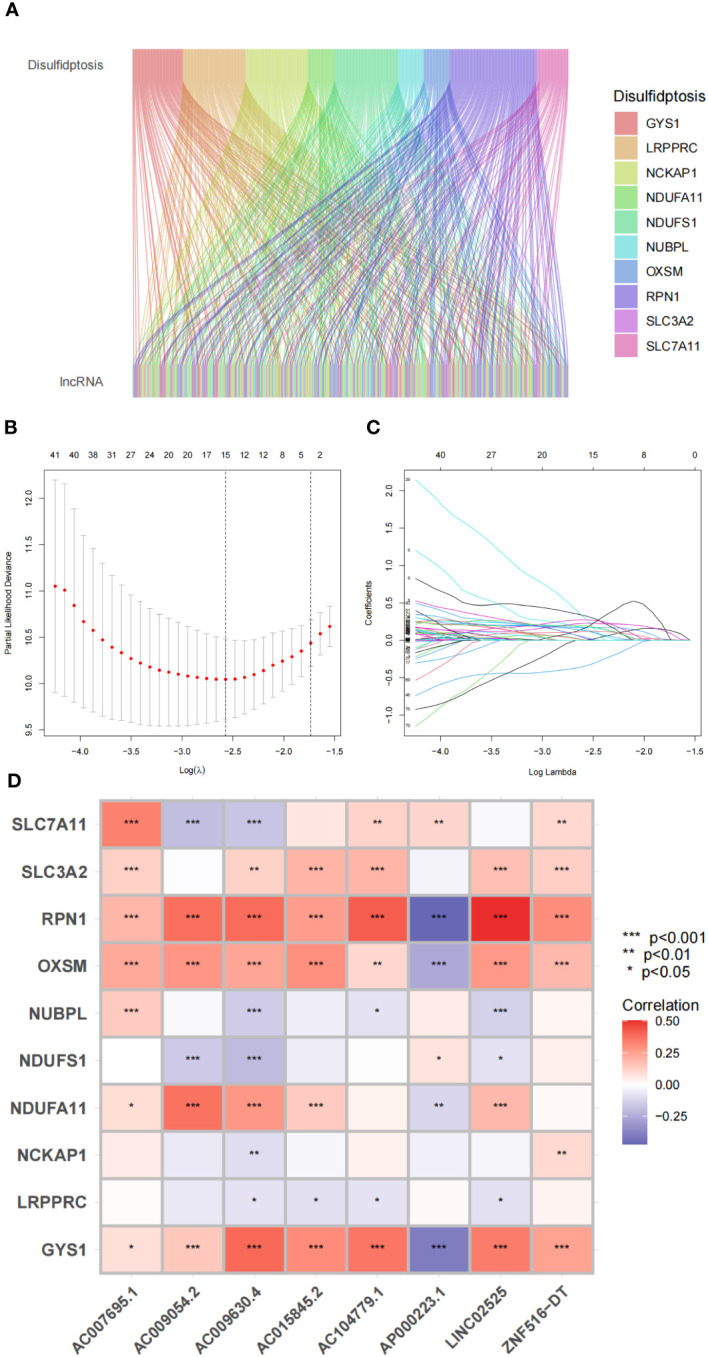
Disulfidptosis-related genes and lncRNA profiles in this study. **(A)** Sankey relation diagram for disulfidptosis genes and lncRNAs. **(B)** The LASSO coefficient profile of disulfidptosis-related lncRNAs. **(C)** The 10-fold cross-validation for variable selection in the LASSO model. **(D)** Heatmap for the correlations between disulfidptosis-related genes and disulfidptosis-related lncRNAs.

We initially screened 261 lncRNAs related to the 10 DRGs from glioma samples in the TCGA database. The direct relationship between DRGs and DRlncRNAs was explained by the Sankey diagram (**Figure 5A**). All datasets were subsequently randomly divided into a training set (276 samples, 50% of total samples) and a test set (275 samples, 50% of total samples). The risk signature was developed using the training set, and their accuracy was verified using the test set. There were no significant differences in clinical traits between the training and test sets (**Table S2**), indicating no bias in clinical traits between the two groups. We employed the multivariate Cox regression analysis and the LASSO regression algorithm identify the optimal combination of DRlncRNAs for prognostic prediction (**Figures 5B, C**). As a result, eight lncRNAs, including *AC007695.1*, *LINC02525*, and *ZNF516-DT*, were selected for inclusion in the construction of the risk signature. Subsequently, we conducted a correlation analysis between the 10 DRGs and these eight modeled lncRNAs, revealing significant correlations between *RPN1*, *OXSM*, and *GYS1* with these lncRNAs (**Figure 5D**).

**Figure 6:**
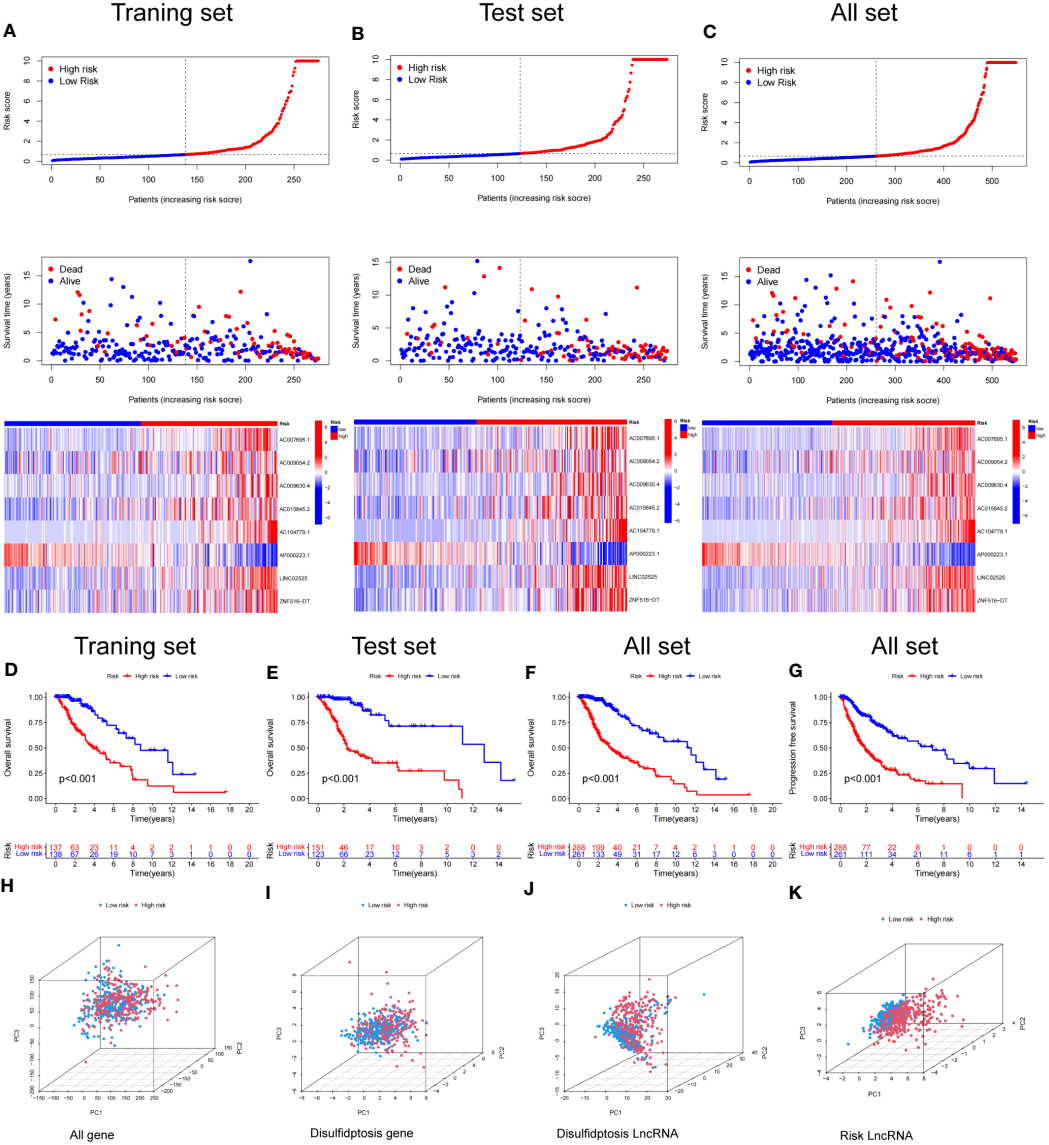
Construction and validation of the prognostic model in the TCGA set. **(A–C)** The disulfidptosis-related lncRNAs signature-based risk score, survival time distributions, and heatmaps were calculated. **(D, E)** The OS analysis between the high-risk and low-risk groups in the training and test sets. **(F)** The OS between the high-risk and low-risk groups in the TCGA cohort. **(G)** The PFS between the high-risk and low-risk groups in the TCGA cohort. **(H–K)** PCA analysis between the high and low-risk based on the whole genome expression set **(H)**, disulfidptosis-related genes **(I)**, disulfidptosis-related lncRNAs **(J)**, and risk model classified by the expression profiles of the 8 disulfidptosis lncRNAs **(K)**.

Based on our signature, we calculated a risk score for each sample. Patients were then divided into high-risk and low-risk groups using the median risk score from the training set as the cutoff. The distributions of risk scores and the expression level of DRlncRNAs in the training set are shown in **Figure 6A**, with the scatterplot distribution of survival times indicating a positive correlation between poorer patient prognosis and the risk score. In addition, we analyzed the risk score, survival time, and expression level of DRlncRNAs in the test set and all sets, with the results of these analyses matching those of the training set (**Figures 6B, C**).

Kaplan–Meier analysis demonstrated that patients in the high-risk group had a significantly worse OS time than those in the low-risk group in the training, test and combined sets (**Figures 6D–F**). Additionally PFS analysis across all sets revealed that patients in LGG with a high-risk classification had a significantly shorter PFS than low-risk patients (**Figure 6G**). These results indicate the robustness of our risk score model in identifying outcomes in LGG patients.

We conducted a principal component analysis to discern the spatial distribution traits of the two subgroups. **Figures 6H–K** sequentially depicts the three-dimensional distribution of the entire gene expression profile, the 10 DRGs, the DRlncRNAs, and the lncRNAs, as per our designated signature. Observations indicate that the low and high‐risk groups, when stratified by DRlncRNAs, exhibited a more scattered distribution than other gene sets.

### Stratified analysis and construction of nomogram diagrams

**Figure 7:**
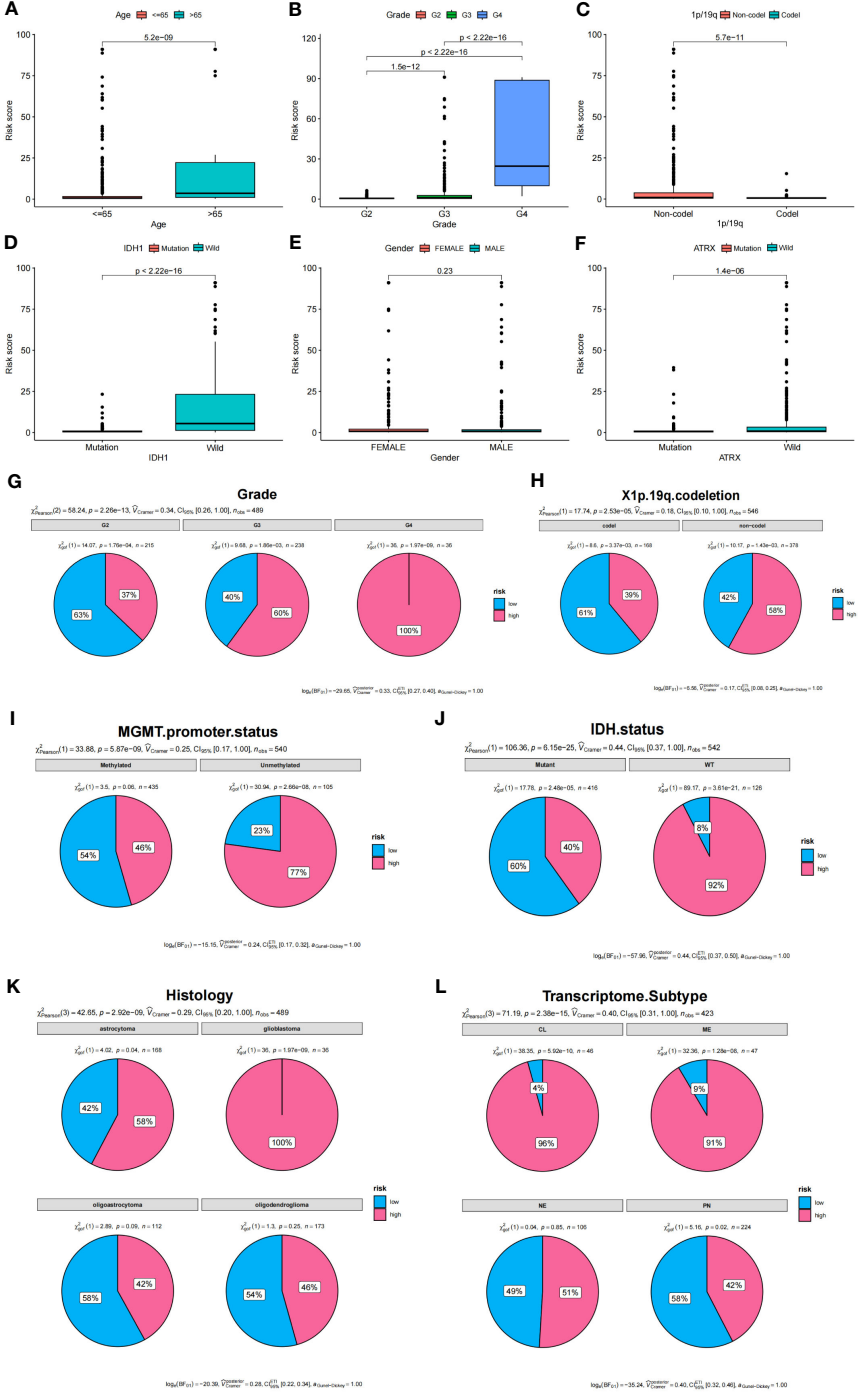
Analysis of clinical features and prognostic risk factors. **(A–F)** Correlations between risk score and clinical features. **(G–L)** Pie diagrams showing the proportion of different risk groups in various clinical features.

To determine the relationship between risk score and clinical characteristics of glioma patients, we analyzed the potential predictors (level of risk, age, gender, WHO grade, 1p/19q, and IDH1 status). Our analysis revealed that, apart from gender and age, clinical grade and IDH1 mutation status were significantly associated with the risk score, while other evaluated clinical characteristics also showed significant associations (**Figures 7A–F**). We further examined the distribution of high and low-risk groups across various clinical features. Pie diagrams revealed that the high-risk group predominantly included clinical features associated with poor prognosis, such as higher grades (Grade3, Grade4), 1p/19q non-codel, unmethylated MGMT promoter, wild-type IDH status, glioblastoma histology, CL and ME Transcriptome subtype (**Figures 7G–L**). Thus, our risk groupings were consistent with existing classifications of clinical features that predicted poorer outcomes.

**Figure 8:**
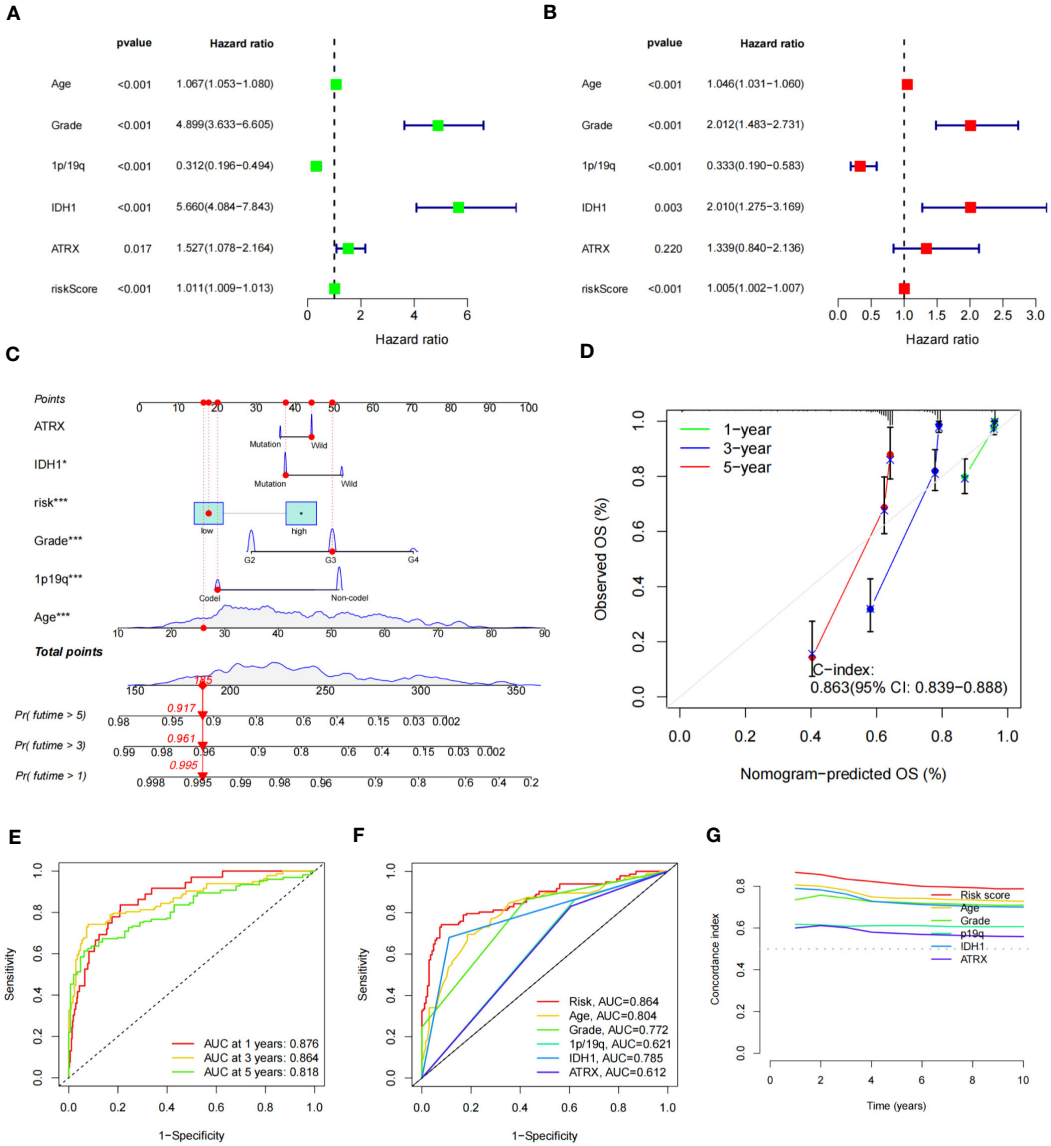
Construction of nomogram of patients with glioma and test of its predictive ability. **(A, B)** Forest plot of univariate **(A)** and multivariate **(B)** Cox regression for prognostic indicators. **(C)** Constructed nomogram for predicting prognosis in glioma patients. The corresponding values of ATRX, IDH1, risk, Grade, 1p19q, and Age were obtained by using their positions at the “Points” abscissa. The total point of a patient is the sum of the four values. And by using the total points coordinate scale, we can get the prognosis prediction of the patient. And the calibration curve of the nomogram reflects the accuracy of nomogram prediction. **(D)** Calibration curves of 1-, 3- and 5-year OS for all glioma patients. **(E)** The AUCs of all sets for 1-, 3-, and 5-year OS rates. **(F)** The prediction accuracy of the nomogram. **(G)** The concordance index analysis of nomogram.

To determine the relationship between the risk score and OS of glioma patients, both univariate and multivariate Cox regression analyses were conducted, focusing on potential OS predictors, including age, grade, 1p/19q status, IDH status, ATRX status, and risk score. Analysis outcomes revealed that the group age over 65 (HR = 1.067, 95% CI = 1.053-1.080, *P<* 0.001), with a higher tumor grade (HR = 4.899, 95% CI = 3.633-6.605, *P<* 0.001), 1p/19q non-codel status (HR = 0.312, 95% CI = 0.196-0.494, *P<* 0.001), IDH1 wild type (HR = 5.660, 95% CI = 4.084-7.843, *P<* 0.001), and a higher risk score (HR = 1.011, 95% CI = 1.009-1.013, *P<* 0.001), exhibited a notably reduced OS (**Figure 8A**). The ATRX status also showed an impact (HR = 1.527, 95% CI = 1.078-2.164, *P* = 0.017) in univariate analysis, but no statistical significance was found in the multivariate regression analysis (*P* = 0.220) (**Figure 8B**).

Subsequently, we constructed a nomogram combining IDH1 status, risk score, WHO grade, 1p/19q status, and age to predict OS (**Figure 8C**). The C-index of nomogram-predicted OS was 0.863 (95% CI:0.839-0.888), indicating a relatively accurate prognosis prediction (**Figure 8D**). The AUCs of the nomogram-predicted OS for 1-, 3-, and 5- year survival rates were 0.876, 0.864, and 0.818, respectively, across all sets (**Figure 8E**). ROC curves for clinical traits were generated using the third year as the prediction period. Risk score (AUC=0.864) seemed to predict prognosis more accurately than clinical features such as age (AUC=0.804), grade (AUC=0.772), and 1p/19q (AUC=0.621) (**Figure 8F**). Furthermore, we calculated the concordance index for these clinical features from 1 to 10 years, and the risk score had the largest area under the curve, further demonstrating the importance of our signature prognostic prediction (**Figure 8G**).

### Functional enrichment analysis

**Figure 9:**
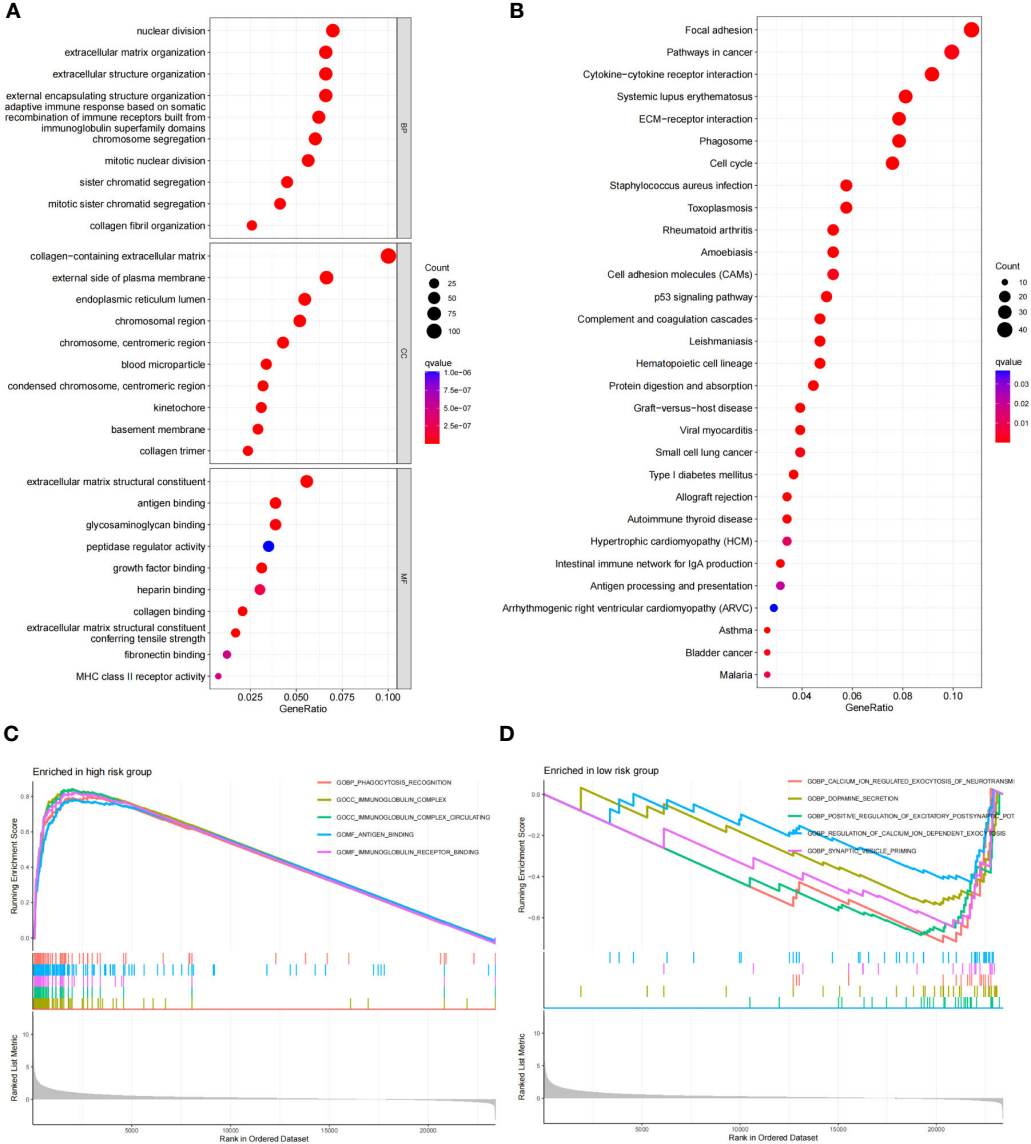
Functional enrichment analysis of risk score differential genes. **(A)** GO of the RDGs signature in the TCGA cohort. **(B)** KEGG of the RDGs signature in the TCGA cohort. **(C, D)** GSEA of the RDGs signature in the TCGA cohort.

Using the criteria of logFCfilter =1 and fdrFilter =0.05, we identified 1406 differentially expressed genes based on our scoring model. Subsequently, we conducted GO, KEGG, and GSEA functional analyses. In the GO analysis, the top three biological processes (BP) were nuclear distribution, extracellular matrix organization and extracellular structure organization (**Figure 9A**). For cellular components (CC), prominent features included the collagen-containing extracellular matrix, the external side of the plasma membrane, and the endoplasmic reticulum lumen. Concurrently, in the molecular function (MF) category, extracellular matrix structural constituent, antigen binding, and glycosaminoglycan binding were notable (**Figure 9A**). The KEGG pathway analysis highlighted significant pathways, especially “focal adhesion” and “pathways in cancers”, explaining the potential tumor regulatory mechanisms (**Figure 9B**). Through GSEA, on comparing the high-risk and low-risk groups, we observed that cytokine-cytokine receptor interaction, ECM receptor interaction, focal adhesion, graft versus host disease and systemic lupus erythematosus were more pertinent to the high-risk group (**Figure 9C**), while, pathways like cardiac muscle contraction, neuroactive ligand receptor interaction, oxidative phosphorylation, Parkinson disease, and ribosome were more related to the low-risk group (**Figure 9D**).

### Correlation analysis of tumor immunity

**Figure 10:**
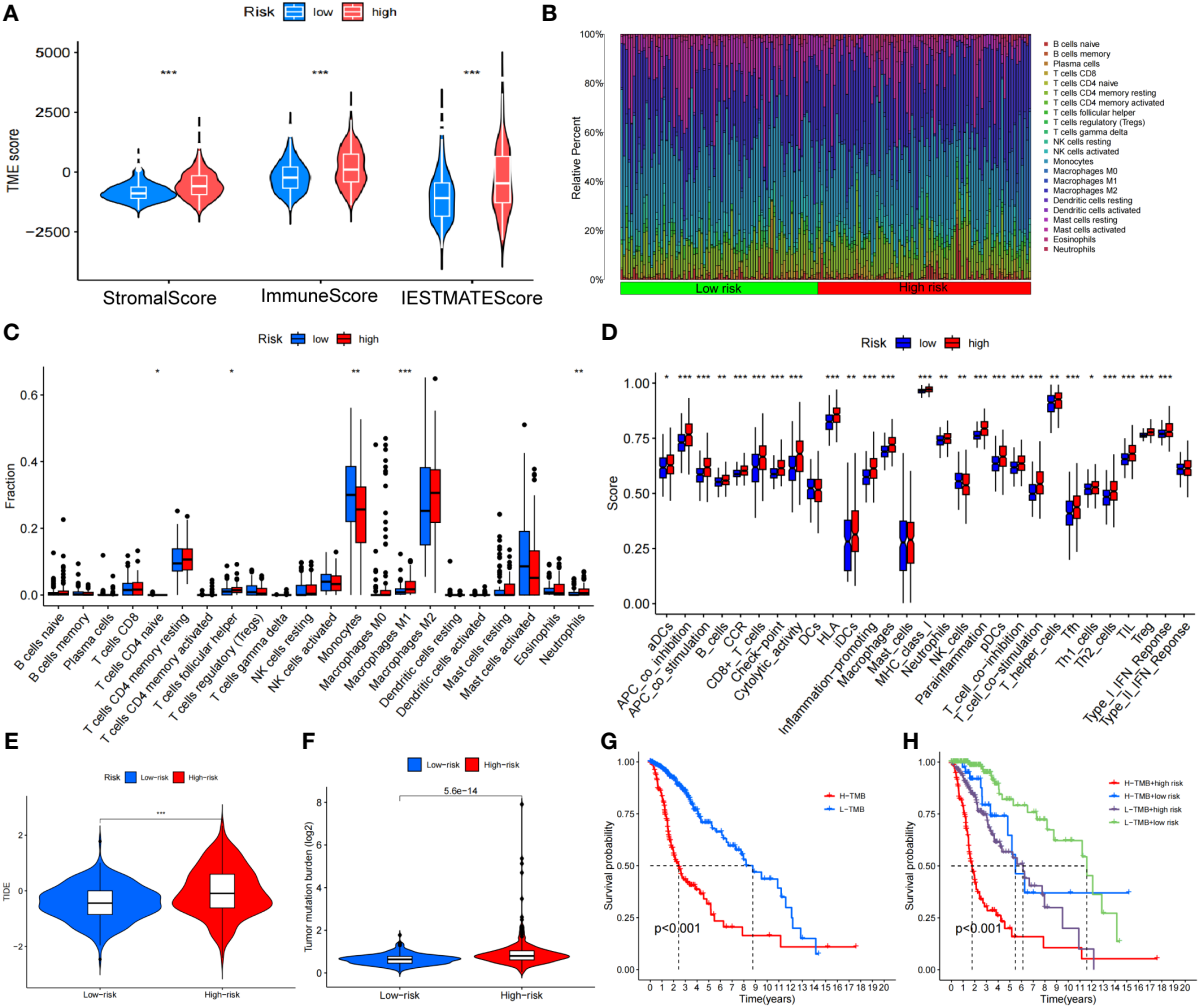
Analysis of the disulfidptosis-related lncRNA prognostic risk score in immune features. **(A)** The assessment of TME-related scores between high- and low-risk groups. **(B, C)** Heatmap of 22 tumor-infiltrating immune cell types in low- and high-risk groups. **(D)** The differences in immune infiltration score between the two groups. **(E)** Differences in TIDE between high and low-risk groups. **(F)** Differences in TMB between high and low-risk groups. **(G)** Kaplan-Meier survival curves of the OS of patients in the high-TMB and low-TMB groups in the entire set. **(H)** Kaplan-Meier survival curves of the OS of patients based on the TMB and risk scores. *P < 0.05, **P < 0.01 and ***P < 0.001.

We calculated the StromlScore, ImmuneScore, and ESTIMATEScore in 676 tumor samples to understand differences in the tumor microenvironment between high- and low-risk groups. The high-risk group exhibited elevated StromlScore, ImmuneScore, and ESTIMATEScore relative to the low-risk group, as depicted in **Figure 10A**. Further examination of immune cell composition revealed a higher fraction of monocytes and neutrophils n the high-risk group (**Figures 10B, C**). In our improved analysis of immune-associated functional disparities, we observed significant differences in functions like APC-co-inhibition, APC-co-stimulation, and B cells among 32 groups when comparing the high- and low-risk cohorts (**Figure 10D**). Additionally, leveraging the TIDE score for each sample to gauge sensitivity to clinical interventions, we discerned that the high-risk group typically displayed higher TIDE scores (**Figure 10E**).

### Tumor mutation burden and drug sensitivity analysis

The TMB level of each sample was calculated based on the total number of molecular mutations after sorting. We first assessed the differences in TMB between the high- and low-risk groups, finding that TMB levels were was significantly higher in the high-risk group compared to the low-risk group (**Figure 10F**). The Kaplan-Meier curve indicated that the high TMB (H-TMB) group had a lower probability of survival (**Figure 10G**). In addition, we performed a combined prognostic analysis for TMB and risk score. The group with low TMB (L-TMB) and a low-risk score demonstrated the highest survival probability (**Figure 10H**). Finally, we screened for potentially sensitive drugs, and the results indicated that 82 drugs might benefit glioma treatment. **Figure S2** displays a selection of these drugs, which could offer insights for potential clinical treatment.

### *LINC02525* was associated with the malignant phenotype of glioma cells

**Figure 11:**
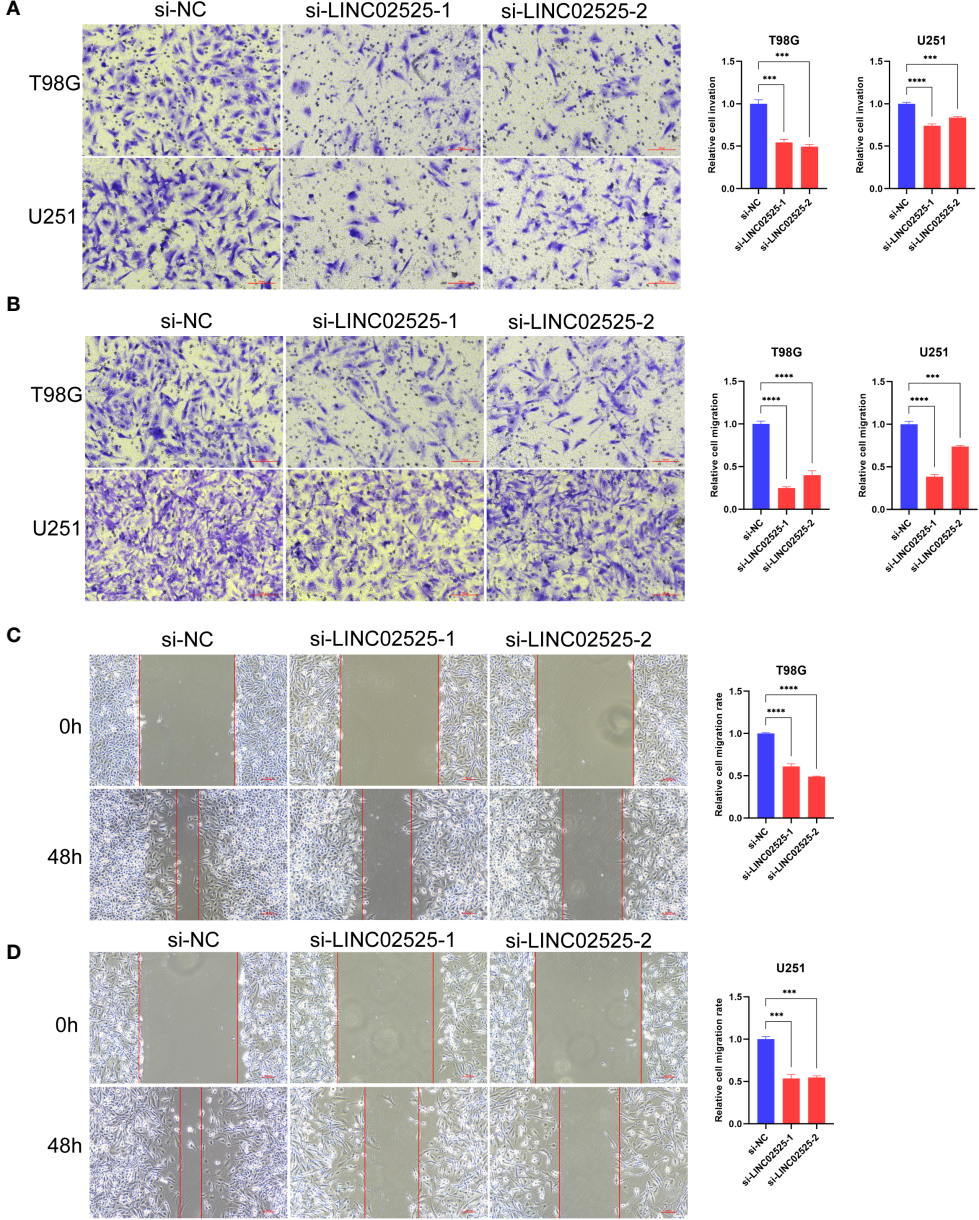
*LINC02525* is associated with the malignant phenotype of glioma cells. **(A)** Transwell assays indicated the invasive abilities of T98G and U251 cells after knocking down *LINC02525*. **(B–D)** Transwell assays **(B)** and wound healing assays **(C, D)** indicated the migratory abilities of T98G and U251 cells after knocking down *LINC02525*. ***P < 0.001 and ****P < 0.0001

**Figure 12:**
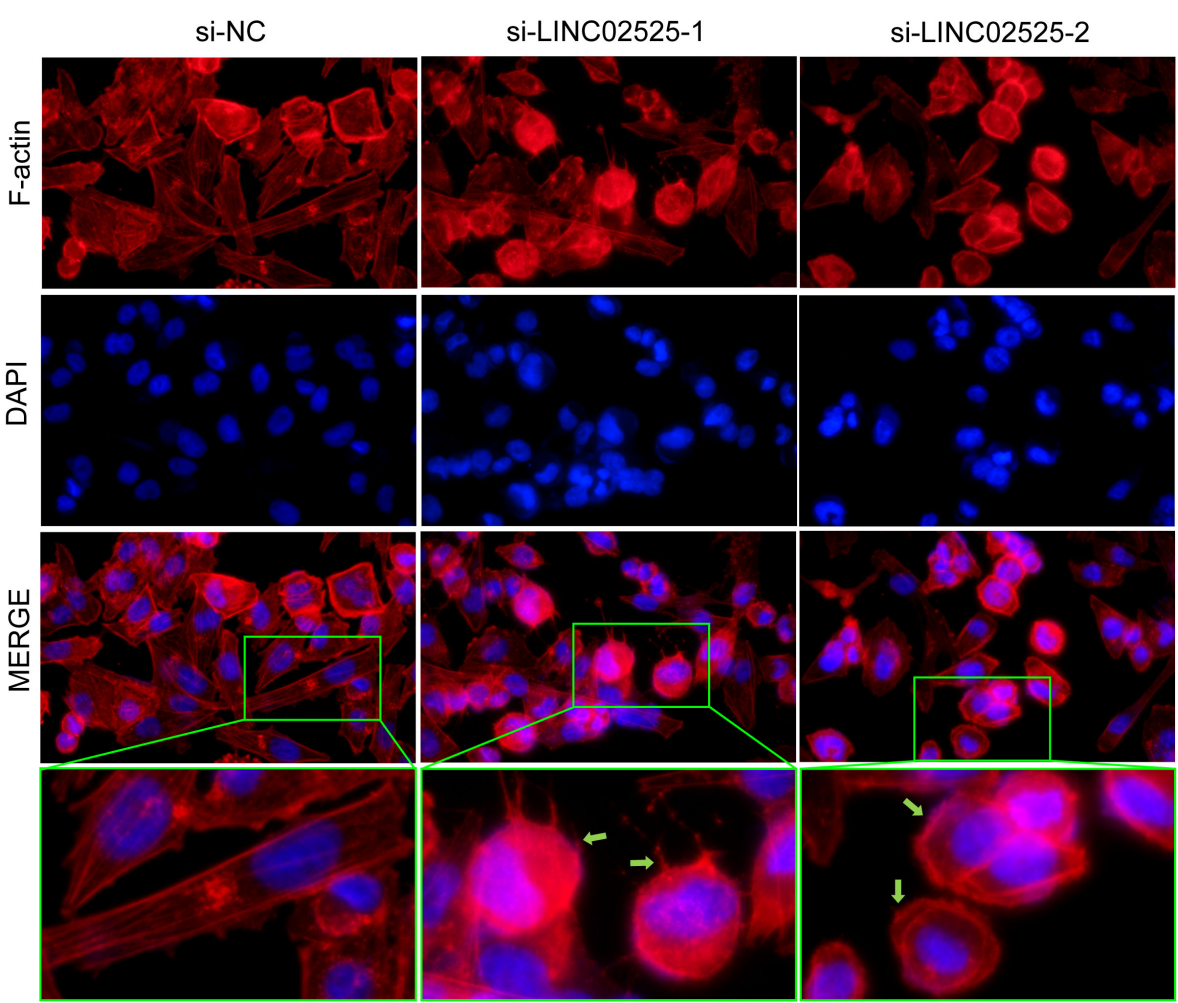
Fluorescent staining of F-actin after knocking down *LINC02525* in the U251 cell line. The green arrows indicate lamellipodia.

We used small interfering RNA (siRNA) to knock down the expression level of *LINC02525* in T98G and U251 cells, **Figure S3** showed knockdown efficiency of LINC02525 expression. Subsequently, transwell assays (**Figure 11A**) revealed a reduction in the invasive abilities of T98G and U251 cells after *LINC02525* expression was knocked down. In addition, **Figures 11B–D** demonstrated that the migration capabilities of T98G and U251 cells decreased after *LINC02525* expression knockdown. Furthermore, knocking down *LINC02525* led to increased actin polymerization, lamellipodia formation, and the development of an actin network cortex branching beneath the cytoplasmic membrane. These changes promote the disulfidptosis of F-actin (**Figure 12**).

## Discussion

Glioma is a highly malignant and heterogeneous neuroepithelial tumor of the brain and spinal cord (29). Owing to its high recurrence rate and the significant disability it causes, intracranial glioma has garnered considerable attention in cancer research (30, 31). In 2021, the World Health Organization updated its neuropathology and molecular pathology diagnostic procedures to offer a more standardized and integrated approach to glioma treatment (7). With the advancement of second-generation sequencing technology and the continuous development of the molecular database, an increasing number of scholars are not only focusing on the identification of molecular subtypes but also investigating the role of programmed cell death in the occurrence and development of malignant tumors. Ferroptosis is an iron-dependent programmed death that is caused by the rupture of the plasma membrane due to excess lipid peroxidation (32). KRAS mutations in pancreatic cancer are often associated with Erastin, a ferroptosis activator (33). Additionally, the tumor suppressor gene P53 can regulate cell tolerance to ferroptosis through different pathways (34, 35). In glioma, ferroptosis may regulate tumor progression by inducing activation and infiltration of immune cells (36). Pyroptosis is another form of programmed cell death, characterized by cell swelling and cytoplasmic efflux. It is triggered by the CASP caspase family in response to infections (37). CASP1-dependent pyroptosis promotes the growth of pancreatic cancer cells when induced by macrophage stimulator factors (38). However, it can also suppress cancer in certain tumors, such as hepatocellular carcinoma(39). Disulfidptosis, on the other hand, is different from other cell death mechanisms as it is related to the stability of the actin cytoskeleton, an essential cell structure that maintains the shape and survival of cells. In glucose-deficient tumor cells, the accumulation of disulfide molecules can result in atypical disulfide bonding among actin cytoskeletal proteins. This, in turn, can lead to the breakdown of the actin network and eventual cell death (18). The definition of disulfidptosis provides new insights into the treatment of tumors. Therefore, we conducted a pan-cancer analysis of disulfidptosis-related genes to investigate their expression in multiple tumors.

*SLC7A11*, *SLC3A2*, *RPN1*, *NCKAP1*, *GYS1*, *OXMS*, *NDUFS1*, *NDUFA11*, *NUBPL* and *LRPPRC* were identified as the 10 genes most associated with disulfidptosis by genome-wide Crispr-Cas9(18). Our subsequent pan-cancer analysis unveiled aberrant expressions of these DRGs across 34 tumor types, including significant occurrences in LGG and GBM. This underscores the pivotal role disulfidptosis may have in tumor progression. The tumor microenvironment serves as a supporting environment essential for tumor cell growth and survival (40). We conducted a pan-cancer analysis to investigate the correlation between disulfidptosis and the tumor microenvironment score. The results indicated diverse immune correlations, suggesting that the influence of these genes on the immune response might vary across different tumor types. In glioma, *RPN1*, *NDUFA11*, and *GYS1* were significantly positively correlated with StromalScore, ImmuneScore, and ESTIMATEScore. Delving deeper into the correlation between DRGs and different immune cells in glioma, we found that *SLC3A2*, *RPN1*, *OXSM*, *NDUFA11* and *GYS1* showed an obvious positive correlation with macrophages M2, which was exactly consistent with what was described above. Our own IHC results also confirmed this.

Tumor-associated macrophages (TAMs) are considered major infiltrating immune cell groups in the tumor microenvironment and have been shown to interact with glioma cells to promote the occurrence and progression of glioma (41, 42). Macrophages are divided into two types according to their activation state and function, namely classically activated macrophages (M1 type macrophages) and alternatively activated macrophages (M2 type macrophages) (43). The M1 macrophages elicit pro-inflammatory role while the latter plays an anti-inflammatory role (44). M2-type macrophages secrete chemokines such as CCL-17, CCL-22 and CCL-24 to recruit Th-2 cells and Tregs, which play an immunosuppressive role. They also secrete anti-inflammatory agents such as IL-4, IL-10 and TGF-β, which further promote tumor development, induce angiogenesis, and inhibit T-cell anti-tumor responses (45). Therefore, M2 macrophages have emerged as indicators of poor prognosis. In our evaluation of the risk factors of eight genes, we identified *SLC3A2*, *RPN1*, *NDUFA11*, *GYS1* and *OXSM* as potential risk factors for glioma prognosis. Notably, *RPN1* and *GYS1* were paramount, consistent with our prior analysis linking them with M2 macrophages. These results further confirm the potential role of disulfidptosis in the development and prognosis assessment of glioma.

Constructing a prognostic risk signature can effectively evaluate the role of multiple genes in tumors and predict prognosis (46). Due to the relatively small number of reported DRGs, we selected 261 lncRNAs closely related to them to construct a prognostic risk signature. Despite having limited protein-coding potential, lncRNAs play a crucial role in cell regulation and are involved in drug resistance to cancer, making them a promising therapeutic option for improving patient prognosis (47). Eight lncRNAs were selected by regression analysis to construct the risk model, and their prognostic prediction was verified through multiple aspects. WHO grade, IDH, and 1p/19q are the main criteria used to determine the prognosis of glioma. Our results confirmed that a high-risk designation is significantly associated with a higher WHO grade, IDH1 wild-type status, absence of 1p/19q codeletion, and ATRX wild-type status, all of which often represent a poor prognosis. We also conducted functional enrichment analysis, which revealed that important pathways such as extracellular matrix organization, extracellular structure organization, focal adhesion, and cytokine-cytokine receptor interaction were involved. These pathways may be related to the involvement of disulfidptosis in the stability of skeleton proteins.

We delved deeper to analyze the correlation between our prognostic signature and the immune dynamics within glioma. The results revealed a significant correlation between our signature scores and measures of the tumor microenvironment, immune cell composition, and overall immune function. The final TIDE score calculation also indicated that the high-risk group may have poor sensitivity to immunotherapy. The TIDE core, a new method to evaluate the mechanism of tumor immune escape, will better help oncologists predict the effectiveness of targeted therapy against immune checkpoints (48). TMB- is the number of errors in the gene code detected per million bases (49). TMB serves as a novel marker to evaluate the efficacy of PD-1 antibodies, as demonstrated in colorectal cancer treatments (50). Our analysis discerned an elevated TMB score within the high-risk patient group, offering insight into potential immunotherapeutic strategies. In addition, our scoring matrix identified 82 potential drug candidates, offering more clinical therapeutic options, but their anti-tumor efficiency needs further experimental verification.

Given the functional enrichment analysis’s emphasis on the “extracellular matrix” - a complex structural entity surrounding and supporting cells within mammalian tissues - we hypothesized that DRlncRNAs might influence tumor cell invasion and migration by modulating glioma cell adhesion to this matrix. Based on this hypothesis, we selected *LINC02525* for experimental validation, as previous studies linked it with neuroblastoma tumor progression (51). After silencing *LINC02525* expression, the invasive and migratory capacities of T98G and U251 cells were notably reduced. This implies that *LINC02525* could enhance glioma’s malignant progression, subsequently influencing patient prognosis. Moreover, silencing *LINC02525* triggered disulfidptosis in F-actin. This observation suggests an association between the DRlncRNAs *LINC02525* and glioma cell disulfidptosis, highlighting its potential as a therapeutic target for glioma.

In conclusion, our findings underscore the multifaceted role of disulfidptosis in glioma progression. Our pan-cancer analysis of DRGs revealed distinct genes that exhibit significant associations with glioma immunity and prognosis. Utilizing DRlncRNAs, we constructed a prognostic risk assessment signature. This model not only accurately forecasts glioma patient outcomes but also sheds light on the mechanisms underpinning disulfidptosis. Furthermore, our results demonstrated that *LINC02525* promotes glioma cell migration and invasion. On the other hand, silencing *LINC02525* augmented disulfidptosis *in vitro* glioma cell experiments. Nevertheless, our study presents several limitations. One major constraint is the unverified specific mechanism of the ten genes associated with disulfidptosis in glioma. Our prognostic risk signature also warrants validation across diverse databases and a broader array of clinical samples. Furthermore, the molecular functions of DRlncRNAs in gliomas remain under-explored, necessitating comprehensive *in vivo* and *in vitro* experimentation. In light of these gaps, our team intends to undertake additional cellular and molecular studies to elucidate disulfidptosis mechanisms in glioma.

## Data availability statement

The original contributions presented in the study are included in the article/**Supplementary Materials**, further inquiries can be directed to the corresponding author/s.

## Ethics statement

The studies involving humans were approved by the Ethics Committee of Xiangya Hospital. The studies were conducted in accordance with the local legislation and institutional requirements. The human samples used in this study were acquired from primarily isolated as part of your previous study for which ethical approval was obtained. Written informed consent for participation was not required from the participants or the participants’ legal guardians/next of kin in accordance with the national legislation and institutional requirements.

## Author contributions

YG: Investigation, Writing – original draft, Writing – review & editing. ZJ: Writing – original draft, Writing – review & editing. QC: Data curation, Writing – review & editing. DX: Data curation, Writing – review & editing. YZ: Formal Analysis, Methodology, Writing – review & editing. WY: Writing – original draft, Writing – review & editing. ZW: Formal Analysis, Methodology, Writing – review & editing. BW: Conceptualization, Project administration, Writing – review & editing. CR: Conceptualization, Project administration, Writing – review & editing. XJ: Conceptualization, Funding acquisition, Supervision, Writing – review & editing.

## Supplementary material

Supplementary Figure 1 Protein expression levels of disulfidptosis-related genes in glioma tissue samples. In The Human Protein Atlas, IHC showed the protein expression level of disulfidptosis-related genes in normal tissue, low-grade glioma, and high-grade glioma samples.

Supplementary Figure 2 Drug sensitivity analysis between high and low-risk groups.

Supplementary Figure 3 Knockdown efficiency of *LINC02525* expression. The knockdown efficiency of *LINC02525* in U251 and T98G cell lines by qPCR.
