# Supplementary material for: Construction and experimental validation of a signature for predicting prognosis and immune infiltration analysis of glioma based on disulfidptosis-related lncRNAs
Source: Front Immunol. 2023 Nov 3;14:1291385. doi: 10.3389/fimmu.2023.1291385 (PMC10655028; doi:10.3389/fimmu.2023.1291385)
Supplement: Supplementary file 2 [file DataSheet_2.pdf]

Table S2. Clinicopathologic characteristics of different sets

	Type	Total set<br>n (%)	Test set<br>n (%)	Training set<br>n (%)	*P* value
Age	<=65	489(91.23)	243(90.67)	246(91.79)	0.76
	>65	47(8.77)	25(9.33)	22(8.21)	
Gender	Female	240(44.78)	121(45.15)	119(44.4)	0.9308
	Male	296(55.22)	147(54.85)	149(55.6)	
Grade	G2	241(44.96)	113(42.16)	128(47.76)	0.2856
	G3	258(48.13)	133(49.63)	125(46.64)	
	G4	37(6.9)	22(8.21)	15(5.6)	
1p/19q	Codel	368(68.66)	179(66.79)	189(70.52)	0.438
	Non-codel	167(31.16)	88(32.84)	79(29.48)	
	unknow	1(0.19)	1(0.37)	0(0)	
IDH1	Mutation	388(72.39)	189(70.52)	199(74.25)	0.3846
	Wild	148(27.61)	79(29.48)	69(25.75)	
ATRX	Mutation	176(32.84)	78(29.1)	98(36.57)	0.0805
	Wild	360(67.16)	190(70.9)	170(63.43)	
