# Supplementary figures and images for: Construction and experimental validation of a signature for predicting prognosis and immune infiltration analysis of glioma based on disulfidptosis-related lncRNAs
Source: Front Immunol. 2023 Nov 3;14:1291385. doi: 10.3389/fimmu.2023.1291385 (PMC10655028; doi:10.3389/fimmu.2023.1291385)

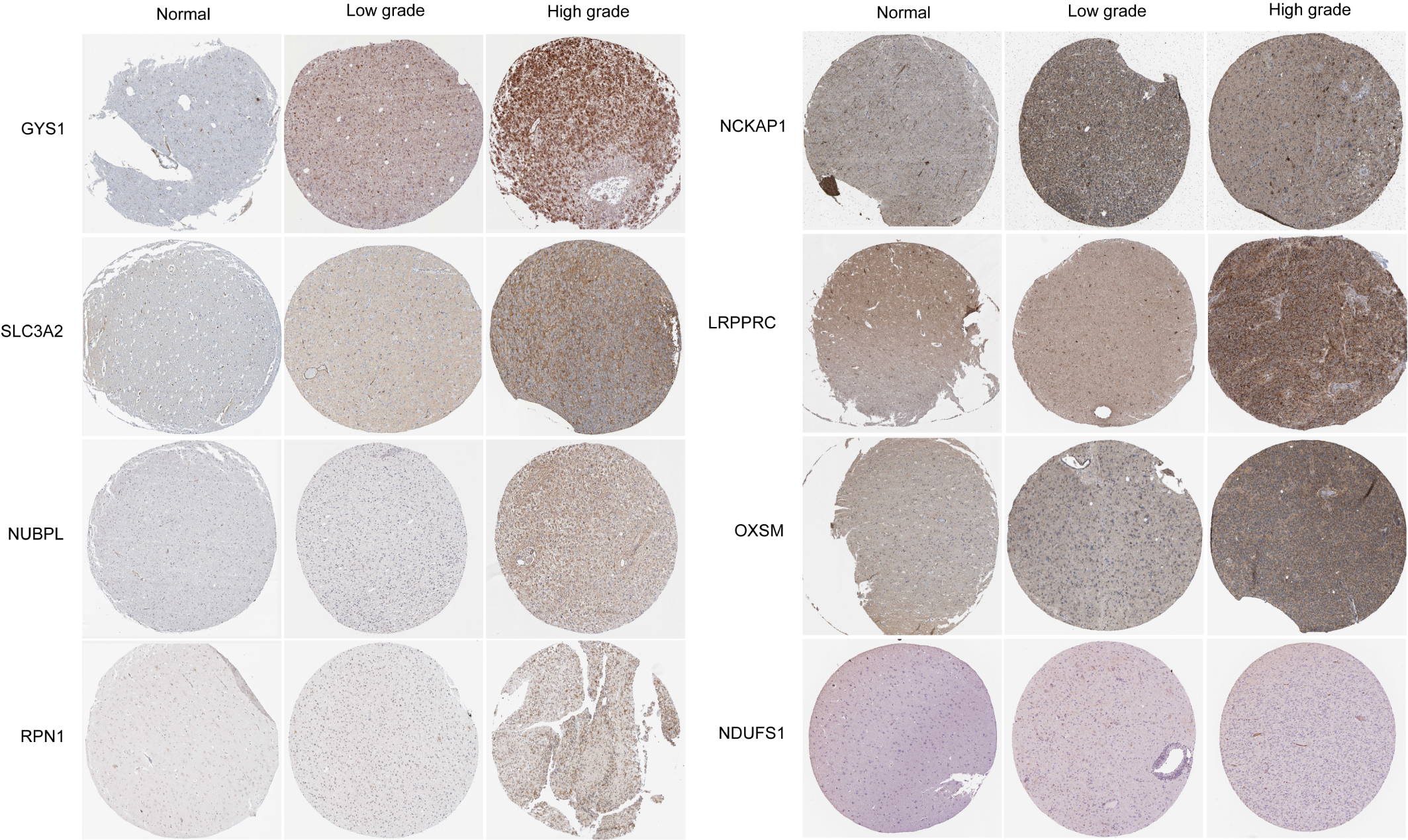

Supplement: Supplementary Figure 1 — Protein expression levels of disulfidptosis-related genes in glioma tissue samples. In The Human Protein Atlas, IHC showed the protein expression level of disulfidptosis-related genes in normal tissue, low-grade glioma, and high-grade glioma samples. [file Image_1.tif]

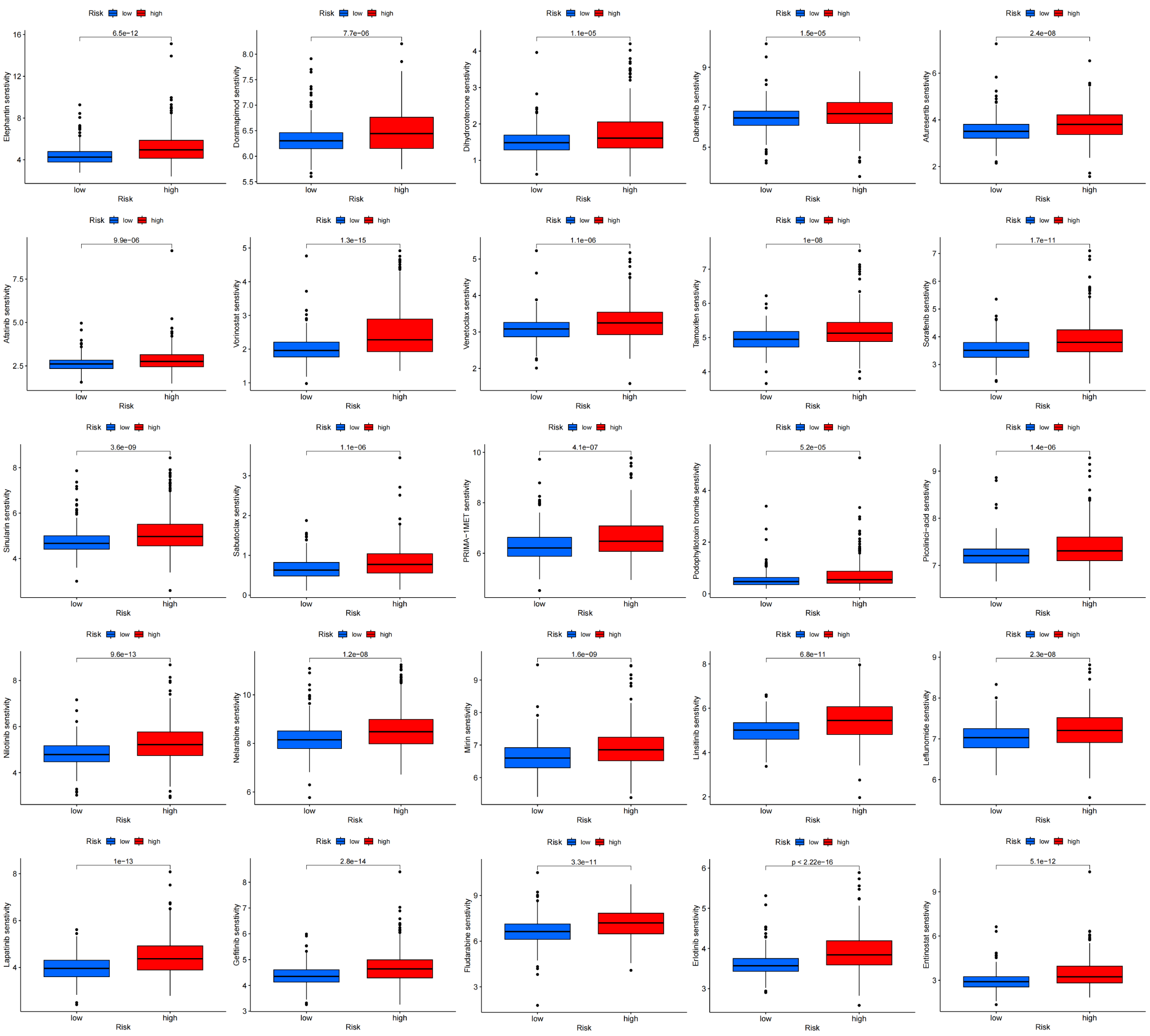

Supplement: Supplementary Figure 2 — Drug sensitivity analysis between high and low-risk groups. [file Image_2.tif]

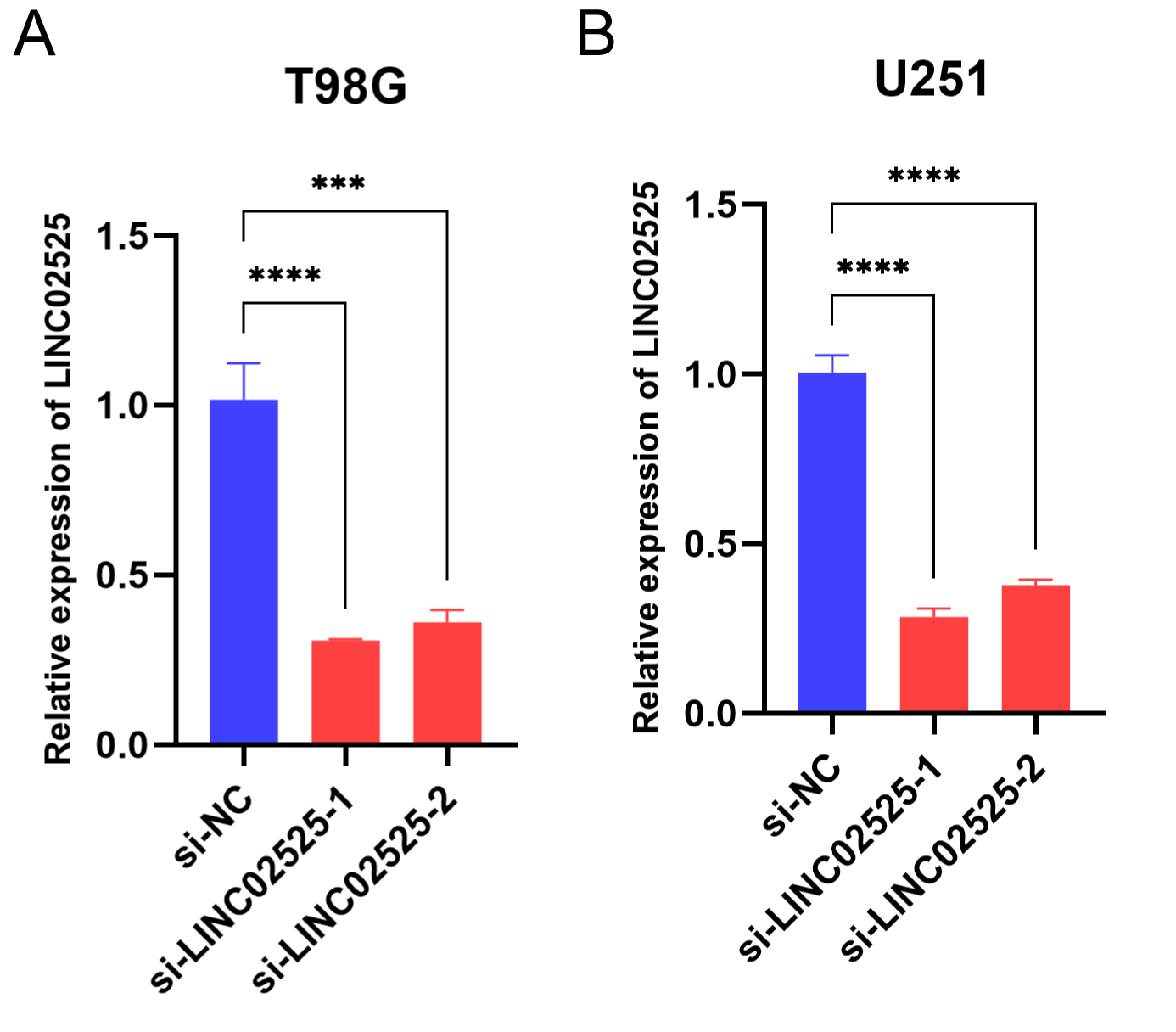

Supplement: Supplementary Figure 3 — Knockdown efficiency of LINC02525 expression. The knockdown efficiency of LINC02525 in U251 and T98G cell lines by qPCR. [file Image_3.tif]
